# From hypoxia to thrombosis: a hypothesis-driven framework for pulmonary embolism risk in deep underground environments

**DOI:** 10.3389/fpubh.2026.1908653

**Published:** 2026-10-22

**Authors:** Yu Mingjing, Huang Yuxi, Hou Wenbo, Deng Mingyuan, Hao Xiangnan, Zhou Haixia, Zhao Shuang

**Affiliations:** 1 Department of Pulmonary and Critical Care Medicine, State Key Laboratory of Respiratory Health and Multimorbidity, West China Hospital, Sichuan University, Chengdu, Sichuan, China; 2 Department of Nursing, Jiamusi College, Heilongjiang University of Chinese Medicine, Jiamusi, Heilongjiang, China; 3 Department of Clinical Medicine, Zhongshan College of Dalian Medical University, Dalian, Liaoning, China; 4 West China School of Medicine, Sichuan University, Chengdu, Sichuan, China

**Keywords:** deep underground environment, hypoxia, occupational exposure, occupational health, pulmonary embolism, risk prediction, thrombosis, venous thromboembolism

## Abstract

Pulmonary embolism (PE) is one of the most severe manifestations of venous thromboembolism, yet current diagnostic and preventive frameworks have largely been developed in ground-based clinical settings. Deep underground environments represent complex occupational exposure systems characterized by relative hypoxia or oxygen fluctuation, normobaric to mildly hyperbaric pressure, high humidity and heat, airborne particulates, radon accumulation, restricted ventilation, limited mobility, circadian disruption, and enclosed psychosocial stress. These factors may interact dynamically with oxygen sensing, cardiopulmonary compensation, venous return, endothelial function, and coagulation–inflammation pathways. However, the relationship between deep underground exposure, thrombogenesis, PE recognition, and prevention remains insufficiently conceptualized. Direct epidemiological evidence linking deep-underground occupational exposure to incident venous thromboembolism or PE remains scarce, and existing PE prediction tools have not been specifically validated in deep-underground occupational populations. Therefore, this review does not assume that underground exposure independently increases PE incidence or that established clinical tools are inadequate. Instead, we develop a hypothesis-driven framework to examine whether exposure-related factors—including oxygen fluctuation, heat and dehydration, restricted mobility, altered hemodynamics, and inflammatory stress—could modify thrombotic susceptibility, symptom interpretation, or the operational performance of conventional PE pathways. We summarize the environmental and physiological stressors relevant to PE, examine how they may amplify Virchow’s triad, and discuss research questions concerning symptom recognition, anticoagulation, reperfusion decision-making, preventive interventions, and the staged development of dynamic risk warning models. This review aims to provide a conceptual foundation for future surveillance, prevention, and clinical research on PE in deep underground occupational populations.

## 1 Introduction

Pulmonary embolism (PE) is a life-threatening manifestation of venous thromboembolism (VTE) and remains a major challenge in clinical medicine and public health. Over the past decades, substantial progress has been made in the diagnosis and management of PE. Most contemporary diagnostic pathways are built on ground-based clinical scenarios, combining pre-test probability assessment, D-dimer testing, computed tomographic pulmonary angiography, anticoagulation, and reperfusion therapy for selected high-risk patients. These approaches have provided a structured framework for emergency departments and hospital-based care. However, they were developed primarily for conventional environments in which patients can access timely laboratory testing, imaging, specialist evaluation, and escalation of treatment. Whether the same conceptual framework is sufficient for extreme occupational settings remains uncertain.

In this review, “deep-underground environments” refer to occupational settings substantially below ground in which workers experience repeated or prolonged exposure to enclosed or semi-enclosed conditions, constrained ventilation or evacuation, and potentially relevant stressors such as oxygen fluctuation, heat and humidity, airborne pollutants, pressure variation, restricted mobility, fixed posture, or prolonged shifts. The framework primarily applies to underground miners, tunnel construction and maintenance workers, and personnel in comparable subterranean engineering or research facilities, rather than short-duration users of well-ventilated underground public spaces. Because these settings vary considerably in depth, oxygen availability, pressure, thermal conditions, ventilation, workload, posture, and exposure duration, they are not treated as a uniform exposure category; instead, the individual exposure dimensions are considered separately as measurable variables (1, 2).

At present, deep underground medicine and PE research have largely developed along separate lines. Studies of deep underground environments have mainly focused on environmental monitoring, cellular stress responses, metabolism, psychological adaptation, fatigue, sleep disturbance, and general occupational health outcomes. In contrast, PE research has concentrated on diagnosis, risk stratification, anticoagulation, reperfusion therapy, and prognosis in ground-level clinical populations (3, 4). This separation has left an important question insufficiently addressed: when PE occurs in deep underground workers, should it be interpreted simply as conventional PE occurring in a special workplace, or might the deep underground environment itself reshape the conditions under which thrombosis develops, is recognized, and is managed?

This question is particularly important because PE risk in deep underground settings may be difficult to capture using traditional clinical reasoning alone. Conventional PE assessment usually begins when symptoms appear or when established risk factors are identified. In deep underground settings, however, symptoms such as dyspnea, chest tightness, tachycardia, fatigue, reduced exercise tolerance, or dizziness may overlap with environmental discomfort, heat stress, hypoxia adaptation, dehydration, or work-related exhaustion. At the same time, cumulative exposure-related stressors may progressively shift workers toward a prothrombotic state before a classical clinical picture of PE becomes apparent (5, 6).

The most fundamental gap is epidemiological. Reliable estimates of VTE or PE incidence among deep-underground occupational populations are currently unavailable, and existing clinical prediction tools have not been specifically validated in these populations. Consequently, it remains unknown whether deep-underground exposure independently modifies PE risk, whether established predictors retain similar calibration and discrimination, or whether exposure-related variables provide incremental predictive value beyond conventional clinical factors. Accordingly, the present review should be interpreted as hypothesis-generating rather than as evidence that current PE frameworks fail in underground workers. Traditional risk factors such as immobilization, tachycardia, dyspnea, hemoconcentration, or inflammatory activation may acquire different meanings in this setting because they may arise from environmental exposure as well as from thromboembolic disease itself (7–9). Similarly, diagnostic delay and limited evacuation capacity may change the consequences of missed or late-recognized PE (4). These considerations raise the possibility that treating the deep-underground environment solely as a passive background may overlook exposure-related modifiers that warrant direct investigation.

In this review, we propose that deep underground environments may constitute a specific risk ecology for PE, shaped by hypoxic sensing, hemodynamic imbalance, venous stasis, endothelial activation, and coagulation–inflammation coupling. This hypothesis does not imply that deep underground exposure has been proven to directly cause PE; rather, it provides a conceptual framework for organizing indirect evidence from deep underground medicine, hypoxia physiology, occupational health, thrombosis biology, and PE management (1, 3, 10). Within this framework, failed adaptation to compound underground exposure is considered as a testable mechanism that could modify pulmonary vascular and thrombotic susceptibility, rather than as an established cause or distinct form of PE.

Accordingly, this review aims to reframe PE risk and prevention in deep underground environments from occupational and pathophysiological perspectives. We discuss the environmental and physiological stressors relevant to PE, their potential interactions with thrombogenesis, and the implications for risk recognition, prevention, and management in settings characterized by cumulative exposure, limited monitoring resources, and delayed evacuation. By linking deep underground medicine with PE pathophysiology, this review provides a conceptual basis for future surveillance, mechanistic studies, and prevention-oriented clinical research in deep underground occupational populations.

## 2 Review approach and scope of evidence synthesis

This hypothesis-driven narrative review integrates evidence from deep-underground medicine, occupational health, thrombosis biology, and PE management. PubMed/MEDLINE, Web of Science, and the China National Knowledge Infrastructure (CNKI) were searched from database inception through May 2026. The search combined terms related to deep-underground occupational settings with terms related to hypoxia, hyperbaric exposure, heat and dehydration, restricted mobility, venous thromboembolism, pulmonary embolism, coagulation, endothelial dysfunction, and risk prediction; equivalent Chinese terms were used in CNKI.

Priority was given to studies directly involving deep-underground environments or underground occupational populations. Because direct studies linking deep-underground exposure to VTE or PE are sparse, mechanistically relevant evidence from hypoxic, high-altitude, hyperbaric, confined-environment, and microgravity studies was also considered. Studies were selected when they addressed PE-relevant environmental exposures, cardiopulmonary adaptation, venous stasis, endothelial activation, coagulation–inflammation pathways, symptom interpretation, clinical management, or risk prediction. Such indirect evidence was used as mechanistic analogy to generate testable hypotheses rather than to establish causality, estimate quantitative PE risk in underground workers, or formulate underground-specific clinical recommendations. As this was a narrative rather than systematic review, no quantitative meta-analysis or formal risk-of-bias assessment was performed (Figure 1).

**Figure 1:**
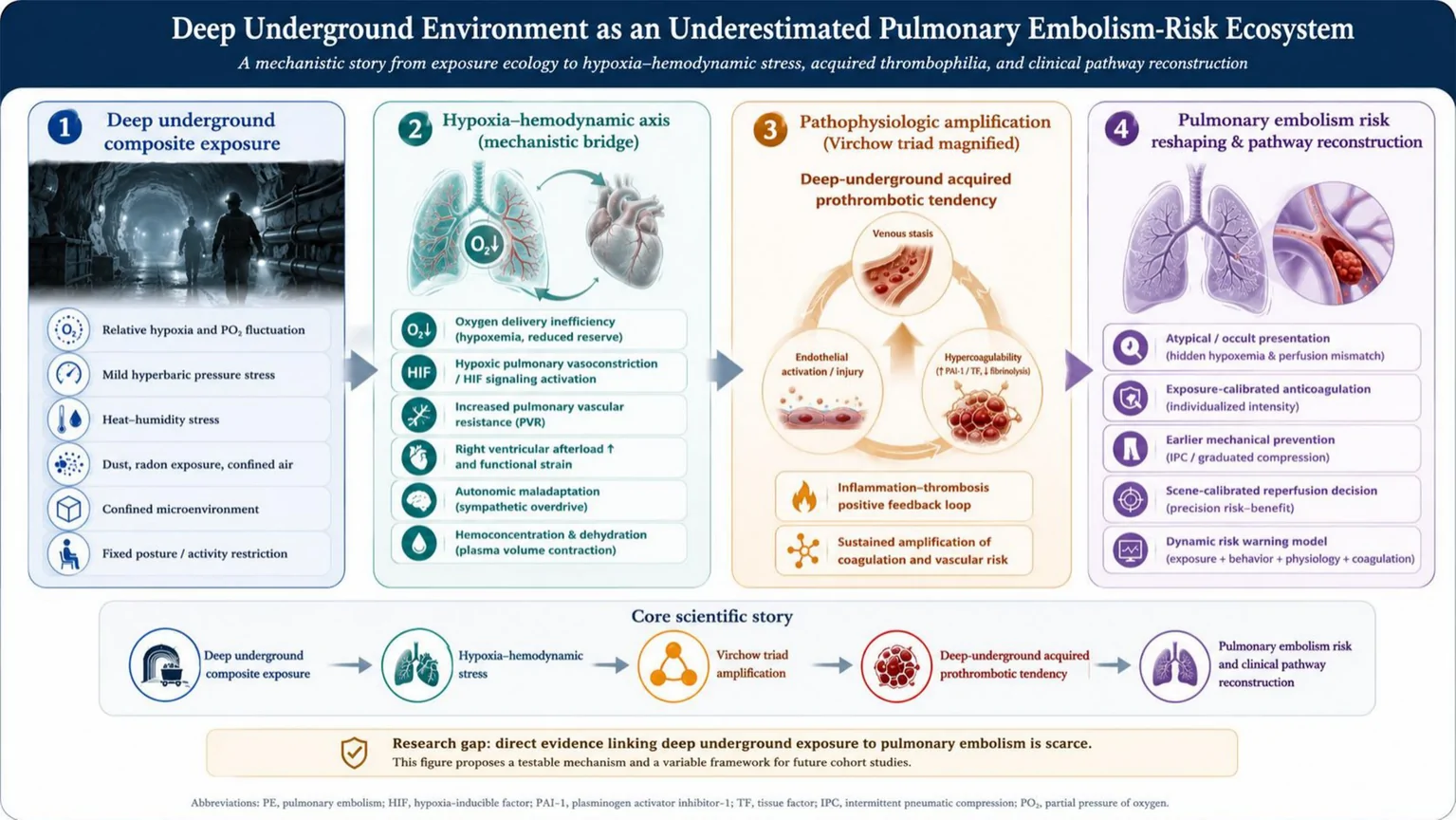
Hypothesis-driven conceptual framework linking deep-underground exposure to potential PE-relevant pathways. Solid elements represent mechanisms supported by general physiological, experimental, or non-underground human evidence, whereas their integration within the deep-underground occupational context remains hypothetical and requires direct epidemiological and prospective validation. The figure should not be interpreted as demonstrating a causal association between deep-underground exposure and incident VTE or PE.

### 2.1 Evidence base and epidemiological uncertainty

A major limitation of this field is the absence of population-based epidemiological data specific to deep-underground workers. Although occupational studies in miners have extensively evaluated respiratory disease, carcinogenic exposures, injury, fatigue, and other health outcomes, we identified no robust cohort data providing incidence or prevalence estimates of VTE or PE specifically among underground miners, tunnel workers, or comparable deep-underground occupational populations. Therefore, it is currently not possible to determine whether PE occurs more frequently in these workers than in matched ground-based populations. The lack of occupation-specific denominator-based data is therefore not evidence of increased risk, but a central knowledge gap that the present framework is intended to define.

### 2.2 Environmental basis: how deep underground exposure creates respiratory–circulatory nonsteady state

The first step in reframing PE risk in deep underground environments is to clarify how underground exposure perturbs respiratory–circulatory homeostasis before thrombosis becomes clinically apparent. This section therefore focuses on the exposure-to-physiology layer: how relative hypoxia or oxygen partial pressure fluctuation, altered pressure, high humidity and heat, restricted ventilation, enclosed microenvironments, and psychosocial stress challenge oxygen transport, pulmonary vascular tone, circulating volume, and autonomic regulation (1, 2, 11–13). These changes do not yet constitute thrombosis, but they may create a high-compensation, low-efficiency physiological state that provides the upstream basis for the prothrombotic mechanisms discussed in the next section (12–16).

#### 2.2.1 Deep underground exposure as a complex stress system rather than an environmental list

The medical relevance of deep-underground work arises not from depth alone, but from the combined and time-varying effects of its environmental and operational exposures. Compared with conventional ground-level workplaces, its defining feature is the cumulative interaction of multiple moderate-intensity stressors rather than an isolated change in oxygen concentration or atmospheric pressure (1, 2).

Among these factors, relative hypoxia or local oxygen partial pressure fluctuation deserves particular attention (14). Deep underground environments are not necessarily continuously hypoxic, and oxygen concentration may remain close to ground-level values when ventilation is adequate (1, 2). Nevertheless, under conditions of restricted ventilation, high worker density, physical exertion, particulate or radon accumulation, and enclosed space exposure, alveolar oxygen partial pressure and tissue oxygen sensing may fluctuate. This distinction is important: for cardiopulmonary physiology, the relevant issue is not whether the environment can be labeled as “hypoxic,” but whether oxygen availability and oxygen sensing are sufficiently disturbed to trigger compensatory responses (15).

Importantly, underground confinement itself should not be assumed to produce adverse physiological effects. In the 40-day Deep Time cavern-isolation experiment, participants who maintained regular daily physical activity showed no detectable structural changes in the major organs and vessels assessed by ultrasound (17). The authors suggested that sustained physical activity and the characteristics of the large-volume cavern environment may have mitigated the physiological effects of confinement. This counterexample suggests that confinement alone is insufficient to support a generalized adverse-effect hypothesis. Any thrombotic effect, if present, would therefore more plausibly depend on the combination of restricted mobility, oxygen disturbance, thermal strain, dehydration, and host susceptibility rather than on underground confinement itself.

The pulmonary circulation is particularly sensitive to oxygen availability. Hypoxia can increase pulmonary vascular tone through hypoxic pulmonary vasoconstriction and hypoxia-inducible factor-related signaling pathways. In the short term, regional hypoxic pulmonary vasoconstriction may help optimize ventilation–perfusion matching. However, sustained or widespread hypoxic exposure may increase pulmonary vascular resistance, elevate pulmonary arterial pressure, and contribute to pulmonary vascular remodeling and pulmonary hypertension (16). Experimental large-animal studies further suggest that hypoxia-induced pulmonary hypertension can impose pressure overload on the right ventricle and initiate compensatory remodeling, including myocardial hypertrophy, inflammatory cell activation, extracellular matrix remodeling, and angiogenic responses (18). Accordingly, oxygen availability represents a biologically plausible upstream component of the proposed hypoxia–hemodynamic axis, although its magnitude and clinical relevance in deep-underground workers remain to be determined.

#### 2.2.2 Reduced oxygen transport efficiency and the transition toward a high-compensation, low-efficiency state

The physiological impact of deep underground exposure can be understood through the framework of the oxygen cascade. Oxygen transport from the external environment to mitochondria requires a sequence of linked processes: pulmonary ventilation, alveolar–capillary diffusion, circulatory oxygen delivery, and tissue oxygen extraction and utilization. Disturbance at any point in this cascade may reduce overall oxygen transport efficiency. Relative hypoxia or oxygen fluctuation first activates peripheral chemoreceptors, leading to hyperventilation and tachycardia to maintain arterial oxygenation and tissue oxygen supply (19). Although this response is initially compensatory, it may also produce respiratory alkalosis, sympathetic activation, and hemodynamic fluctuation (13, 16, 19, 20).

At the circulatory level, hypoxia may activate the HIF–EPO pathway, stimulate erythropoiesis, and increase hemoglobin concentration, thereby improving blood oxygen-carrying capacity (21). However, this adaptation has physiological limits. Excessive erythrocytosis or hemoconcentration may increase blood viscosity, reduce cardiac output, impair microcirculatory perfusion, and create a state in which oxygen-carrying capacity increases but delivery efficiency declines. In parallel, hypoxic pulmonary vasoconstriction may be beneficial when localized, but sustained or diffuse pulmonary vasoconstriction increases pulmonary vascular resistance and right ventricular afterload (5, 15). These processes suggest that the respiratory–circulatory response to underground exposure may not be simply protective; it may become inefficient and vulnerable when compensatory demands are prolonged.

Other underground exposures may further aggravate this imbalance. High humidity and heat increase ventilatory burden and fluid loss, thereby promoting dehydration and hemoconcentration (8, 22). Particulates and radon may contribute to airway and microvascular inflammation or oxidative stress. Enclosed space, fatigue, and psychosocial stress may intensify sympathetic activation and impair autonomic balance (23, 24). Thus, the relevant pathophysiological pattern is not “hypoxia alone,” but a combined state in which oxygen availability, pulmonary vascular resistance, circulating volume, blood viscosity, and autonomic regulation are simultaneously challenged.

Studies conducted under hypoxic and other extreme environmental conditions demonstrate alterations in oxygen transport, vascular tone, and autonomic regulation. Whether the same changes occur to a clinically meaningful extent in deep-underground workers is unknown. We therefore hypothesize that compound underground exposure could create a high-compensation, low-efficiency physiological state in susceptible workers.

#### 2.2.3 Autonomic readaptation disorder as an intermediate link between exposure and thrombotic susceptibility

Nevertheless, indirect evidence from high-altitude hypoxia, hyperbaric exposure, confined environments, and spaceflight analogs suggests that complex environmental exposure can place the autonomic nervous system under sustained regulatory demand (6, 13, 16, 25–27). Hypoxia activates sympathetic outflow through peripheral chemoreceptor stimulation, leading to increased heart rate, reduced high-frequency components of heart rate variability, and an elevated low-frequency/high-frequency ratio. With prolonged exposure, heart rate may partially decline, but it often does not return fully to baseline, suggesting that adaptation may represent a new high-load equilibrium rather than complete normalization (13, 16).

Non-hypoxic stressors may further amplify autonomic imbalance. Heat and humidity increase cardiovascular strain and fluid loss; particulate exposure and impaired air quality may enhance inflammatory and oxidative stress; enclosed environments, sleep disturbance, and psychological pressure may increase fatigue (28) and sympathetic dominance. Hyperbaric chamber studies using photoplethysmographic waveform decomposition have shown that increased pressure is associated with changes in systolic and diastolic waveform parameters, suggesting peripheral vasoconstriction and increased vascular resistance (26). Evidence from diving and underwater exposure also indicates that pressure change, blood oxygen fluctuation, and altered regional perfusion increase cardiovascular autonomic regulatory load. In addition, spaceflight and simulated microgravity studies indicate that confinement, isolation, abnormal atmospheric conditions, and psychological stress may disturb autonomic regulation, with post-exposure orthostatic intolerance partly attributed to altered blood volume status and insufficient autonomic readaptation (27).

For PE risk, this autonomic pathway is important because it may connect environmental exposure with venous and microcirculatory vulnerability. Sustained sympathetic activation and increased peripheral vascular resistance may reduce venous return efficiency and alter hemodynamic stability. Reduced heart rate variability may indicate diminished physiological reserve for buffering hypoxia, dehydration, posture-related venous pooling, and circulatory stress. When these changes coexist with heat-related dehydration, restricted mobility, fixed posture, and inhalational stress, autonomic regulation may become less capable of buffering circulatory fluctuation. This creates a vulnerable hemodynamic state, which is considered in the next section as one of the pathways linking underground exposure to thrombogenesis (13, 16).

In summary, the key stressor defined in this section is not underground location itself, but a hypoxia–hemodynamic axis that links oxygen fluctuation, pulmonary vascular resistance, right ventricular load, autonomic readaptation, venous return, and oxygen-delivery efficiency. This axis does not constitute thrombosis by itself, but it creates the upstream physiological condition under which the prothrombotic pathways discussed below may become amplified (Table 1).

**Table 1:** Candidate exposure variables, evidence status, and monitoring indicators relevant to PE risk in deep-underground environments.

Exposure domain	Evidence status	PE-relevant pathway	Potential consequence	Candidate monitoring indicators
Relative hypoxia or oxygen fluctuation	Indirect mechanistic evidence from hypoxia, high-altitude, experimental, and disease-population studies; underground-specific VTE evidence is absent	HPV; HIF activation; impaired oxygen delivery; increased PVR	RV load; endothelial activation; occult hypoxemia; procoagulant shift	Ambient oxygen partial pressure; SpO₂ trend; respiratory rate; heart rate; HRV; D-dimer; coagulation–fibrinolysis markers
Prolonged fixed posture or restricted mobility	Established physiological basis for venous stasis, but its contribution to VTE in underground workers has not been directly evaluated	Reduced calf muscle pump; venous valve-sinus stasis; slow lower-limb flow	Lower-limb DVT; local venous hypoxia; venous stasis	Posture duration; step count; activity level; wearable posture data; lower-limb venous ultrasound
Heat–humidity stress and inadequate hydration	Supported by general heat-stress and dehydration physiology; underground-specific thrombotic evidence is limited	Fluid loss; plasma volume reduction; hemoconcentration; increased viscosity	Hypercoagulability; impaired microcirculation; dehydration-related risk accumulation	Temperature; humidity; fluid intake; urine output; urine specific gravity; hematocrit; hemoglobin; electrolytes
Airborne particulates, radon, diesel exhaust, or confined air pollutants	Underground occupational exposure is documented, but the proposed link with thrombosis is based mainly on indirect inflammatory and endothelial evidence	Airway inflammation; oxidative stress; endothelial activation; inflammation–coagulation coupling	Endothelial dysfunction; inflammatory prothrombotic activation; symptom overlap with PE	Dust/gas sensors; radon monitoring; respiratory symptoms; CRP; IL-6; endothelial injury markers; SpO₂
Mild hyperbaric pressure or pressure fluctuation	Indirect evidence from hyperbaric, diving, and physiological studies; the direction and clinical relevance of the effect remain uncertain	Altered venous capacitance; central blood-volume shift; vasomotor response; autonomic load	Variable effect on venous return; microcirculatory or venous-flow instability	Ambient pressure; PPG waveform; HRV; blood pressure; lower-limb venous ultrasound
Enclosed psychosocial stress, fatigue, sleep disturbance, or circadian disruption	Limited direct evidence for physiological effects in confined environments; the proposed relationship with VTE remains speculative	Sympathetic activation; reduced HRV; impaired autonomic readaptation; metabolic–inflammatory disturbance	Increased vascular resistance; reduced physiological reserve; stress-related prothrombotic background	Heart rate; HRV; sleep duration/quality; shift pattern; fatigue score; psychological stress scale
Cumulative work duration and repeated exposure	Hypothesis-derived exposure construct without direct validation against VTE or PE outcomes	Time-dependent accumulation of hypoxia, dehydration, immobility, autonomic strain, and inflammation	Exposure-induced prothrombotic window before overt PE symptoms	Time-stamped exposure records; cumulative underground hours; serial physiological or laboratory measurements; interval lower-limb ultrasound assessment in prospective research settings

CRP, C-reactive protein; DVT, deep vein thrombosis; HIF, hypoxia-inducible factor; HPV, hypoxic pulmonary vasoconstriction; HRV, heart rate variability; IL-6, interleukin-6; PE, pulmonary embolism; PPG, photoplethysmography; PVR, pulmonary vascular resistance; RV, right ventricular; SpO₂, peripheral oxygen saturation. Evidence classified as “indirect” was derived from non-underground human populations, experimental models, or analogous extreme environments and should not be interpreted as demonstrating a causal association between deep-underground exposure and VTE or PE. None of the listed variables has been validated as an independent predictor of VTE or PE in deep-underground workers.

### 2.3 Mechanistic reframing: translating respiratory–circulatory nonsteady state into prothrombotic tendency

Building on the respiratory–circulatory nonsteady state described above, this section focuses on how prolonged compensatory stress may be translated into thrombotic susceptibility. Rather than re-listing underground exposures, we examine how impaired venous return, hemoconcentration, endothelial activation, and coagulation–inflammation coupling may remodel Virchow’s triad in deep underground settings. In this framework, thrombosis is not an inevitable consequence of underground work, but a potential downstream outcome when adaptive responses become prolonged, inefficient, and biologically procoagulant.

#### 2.3.1 Systemic amplification of Virchow’s triad under compound deep underground exposure

In the exposure context described above, venous stasis represents the most direct mechanical pathway through which respiratory–circulatory nonsteady state may be converted into thrombosis. Prolonged fixed posture, restricted locomotion, reduced calf muscle-pump activity, heat-related dehydration, and sympathetic-mediated peripheral vasoconstriction may jointly reduce venous return efficiency (11, 29). Thus, venous stasis in underground settings should not be understood only as classical immobilization, but also as posture-related and exposure-accumulated impairment of venous flow.

Endothelial activation may also acquire a broader meaning in this setting. Hypoxia and inhalational exposures, including particulates, radon, diesel exhaust, and blasting-related products, may contribute to oxidative stress and inflammatory responses, which could in turn promote endothelial activation and altered vascular homeostasis (1, 2, 12, 30).

Hypercoagulability may be enhanced when compensatory responses for oxygen delivery interact with fluid loss and impaired microcirculatory perfusion. Increased hemoglobin concentration or hemoconcentration may improve oxygen-carrying capacity in some contexts, but when combined with dehydration and reduced plasma volume, it may increase blood viscosity and favor platelet activation and fibrinolytic imbalance. Accordingly, “deep underground thrombosis” should not be interpreted as a distinct disease entity, but as a remodeling of Virchow’s triad under compound environmental exposure (5, 12, 30).

#### 2.3.2 Hypoxia–HIF-mediated procoagulant activation and the inflammation–thrombosis feedback loop

Beyond its hemodynamic effects, relative hypoxia may directly shift the coagulation–fibrinolysis balance toward thrombosis through hypoxia-inducible factor (HIF)-related pathways. Under hypoxic conditions, the activity of prolyl hydroxylase domain proteins and factor inhibiting HIF is reduced, allowing HIF-1α and HIF-2α to stabilize, translocate into the nucleus, and activate hypoxia-responsive gene transcription. Among HIF-related downstream targets, plasminogen activator inhibitor-1 (PAI-1) and tissue factor (TF) are particularly relevant to thrombosis. Existing experimental and mechanistic studies suggest that hypoxia may increase PAI-1 expression, thereby inhibiting tPA/uPA-mediated fibrinolysis. At the same time, hypoxia may enhance TF-mediated extrinsic coagulation and downregulate anticoagulant factors such as tissue factor pathway inhibitor and protein S, leading to a combined state of enhanced coagulation, weakened anticoagulation, and impaired fibrinolysis (31, 32).

The evidence supporting this mechanism comes from multiple indirect but convergent sources, including hypoxic cell models, animal thrombosis models, ex vivo platelet hypoxia studies, transcriptomic analyses in disease populations, and high-altitude human studies. Chaurasia et al. demonstrated that anucleate human platelets can translate and stabilize HIF-2α under hypoxia, leading to PAI-1 synthesis and extracellular vesicle release; hypoxia-mimetic treatment accelerated thrombosis in mice, and increased platelet HIF-2α and PAI-1 expression was also observed in patients with chronic obstructive pulmonary disease and high-altitude residents (31). Gangaraju et al. found upregulation of F3, SELP, VEGFA, SLC2A1, SERPINE1, and inflammation-related genes in granulocytes and platelets from patients with polycythemia vera or essential thrombocythemia, with some genes showing stronger expression in patients with prior thrombosis, suggesting a link between HIF activity, inflammation, and thrombotic risk (33). Jha et al. further reported that differentially expressed genes in high-altitude deep vein thrombosis were enriched in pathways related to response to hypoxia, coagulation, hemostasis, and platelet activation, indicating that hypoxia may prime a prothrombotic transcriptional background (34).

Hypoxia may further amplify inflammation–coagulation coupling through reactive oxygen species, NF-κB signaling, inflammasome activation, inflammatory cytokines, platelet activation, and neutrophil extracellular traps. Once thrombosis develops, local hypoxia within the thrombotic microenvironment may further sustain HIF activation, tissue factor signaling, impaired fibrinolysis, and inflammatory amplification. This creates a potential hypoxia–inflammation–thrombosis loop, especially when oxygen fluctuation coexists with the exposure-related stressors described above (5, 12).

#### 2.3.3 Mild hyperbaric pressure is not necessarily protective: posture-related venous stasis and the valve-sinus prothrombotic microenvironment

The effect of mild hyperbaric pressure on venous return should not be interpreted as inherently protective. Although mildly increased ambient pressure might theoretically reduce peripheral venous capacitance or shift blood centrally, real-world underground work involves pressure exposure together with restricted space, prolonged static posture, reduced locomotion, heat-related dehydration, oxygen fluctuation, and psychological stress. Under these conditions, pressure-related changes in regional perfusion and blood-volume distribution may coexist with restricted mobility and posture-related venous stasis; however, the net effect on venous return in underground workers remains unknown (35–37).

Evidence from spaceflight and microgravity research supports a broader principle: altered pressure or fluid distribution does not necessarily translate into effective venous drainage (38, 39). A recent review of coagulation under microgravity further supports the view that extreme environmental exposures may influence thrombosis through interacting vascular and coagulation mechanisms rather than through a single hemodynamic pathway. Mayor et al. proposed a two-hit model involving endothelial/glycocalyx dysfunction together with a shift toward procoagulant thrombin generation, potentially related in part to altered fluid and sodium handling. Although microgravity is physiologically distinct from deep-underground exposure, this model is conceptually relevant because it illustrates how abnormal fluid distribution, endothelial perturbation, and coagulation activation may coexist in extreme environments. This analogy should not be interpreted as direct evidence for underground-associated thrombosis (40). Venous dilation, slow flow, stagnation, and even thrombosis have been reported under extreme conditions in which fluid distribution and vascular regulation are profoundly altered. Together, these observations caution against equating central blood-volume redistribution or increased venous filling with effective venous drainage or reduced thrombotic risk. Reports from high-altitude settings further suggest that hypoxia, hemoconcentration, reduced mobility, and host susceptibility may interact to create a prothrombotic state (41–43). In underground workers, we hypothesize that local low-flow states in venous valve sinuses, compression of calf or popliteal veins, hemoconcentration, and endothelial activation may outweigh any theoretical pressure-related improvement in venous return (11, 29, 30).

#### 2.3.4 Applicability of established PE prediction tools: an untested question in deep-underground populations

Current PE clinical prediction rules, including the Wells and Geneva scores, remain the appropriate starting point for PE assessment in underground workers. Importantly, there is currently no evidence demonstrating that these tools perform worse in deep-underground populations, because their discrimination, calibration, and clinical utility have not been specifically evaluated in this setting. The relevant question is therefore not whether these scores are invalid, but whether exposure-related variables modify the interpretation of individual predictors or provide incremental predictive information beyond established clinical factors. These scores were developed for ground-level clinical settings and emphasize previous VTE, malignancy, recent surgery or immobilization, lower-limb symptoms, hemoptysis, and tachycardia (3, 4). In deep-underground environments, several of these variables may acquire different meanings because dyspnea, tachycardia, chest discomfort, and reduced exercise tolerance may arise from PE, but also from oxygen fluctuation, heat stress, dehydration, or sympathetic activation (8, 16, 22).

Similarly, the conventional concept of immobilization may fail to capture posture-related venous stasis caused by narrow workspaces, squatting, prolonged sitting, reduced locomotion, or insufficient calf muscle-pump activity. The key issue is therefore not to abandon Wells or Geneva scores, but to determine whether their performance is modified by the underground context and whether exposure-specific information adds clinically meaningful information beyond these established tools. Candidate exposure variables for future evaluation therefore include exposure duration, oxygen availability, temperature and humidity, hydration status, posture duration, activity level, hemoconcentration, inflammatory or endothelial injury markers, and lower-limb venous ultrasound findings (38, 44–46). Detailed dynamic modeling based on these variables is discussed in Section 4.4. Such variables should only be incorporated into a formal prediction model if prospective studies demonstrate incremental value beyond established predictors, for example through improvement in calibration, discrimination, and clinical decision utility.

Importantly, any putative underground-associated prothrombotic tendency should be interpreted against the background of individual VTE susceptibility rather than as an isolated occupational effect. Age, obesity, previous VTE, malignancy, recent surgery or trauma, hormonal therapy, inherited or acquired thrombophilia, smoking, and relevant cardiopulmonary comorbidities may determine the baseline risk onto which cumulative occupational exposures are superimposed. Repeated underground exposure may therefore act as an effect modifier or additional “hit” rather than a sufficient cause of thrombosis. Future occupational health programs should document baseline VTE risk before or at workforce entry and update transient clinical risk factors longitudinally, while separately recording time-varying occupational exposures such as cumulative underground hours, heat and dehydration burden, oxygen fluctuation, restricted posture, and activity level. Comparing within-worker changes across exposure and recovery periods, together with appropriately selected surface-worker or lower-exposure reference groups, may help distinguish exposure-associated physiological changes from background individual susceptibility.

### 2.4 Prevention and management reframing: from prothrombotic tendency to context-adapted PE intervention

If future studies confirm that deep-underground exposure contributes to prothrombotic susceptibility through cardiopulmonary nonsteady state and Virchow’s triad remodeling, its clinical relevance would lie in how this context modifies PE recognition, monitoring, prevention, and treatment decisions (Table 2).

**Table 2:** Established pulmonary embolism pathways versus potential challenges in deep-underground settings.

Clinical dimension	Conventional ground-based PE pathway	Challenge in deep-underground settings	Questions or candidate strategies for future evaluation
Risk recognition	Wells/Geneva scores; symptom-triggered assessment	Exposure-related risk may accumulate before classical symptoms	Evaluate whether SpO₂, heart rate, HRV, posture duration, hydration status, and work duration provide incremental value for risk recognition
Symptom interpretation	Dyspnea, chest pain, hemoptysis, syncope, tachycardia, hypoxemia	Heat stress, hypoxia adaptation, fatigue, and dehydration may mimic or mask PE	Focus on unexplained desaturation, disproportionate tachycardia, abrupt exercise intolerance, syncope, and new lower-limb symptoms
Diagnostic pathway	D-dimer followed by CTPA when indicated	Imaging and laboratory testing may be delayed by evacuation constraints	Feasibility and diagnostic contribution of portable oximetry, lower-limb ultrasound, bedside echocardiography, or EIT before definitive imaging
Anticoagulation	Standard LMWH or DOAC dosing; routine drug monitoring usually unnecessary	It remains unknown whether hypoxia, inflammation, dehydration, or altered organ perfusion meaningfully affects anticoagulant exposure or bleeding risk.	Whether exposure-related physiological changes meaningfully alter renal/hepatic function, anticoagulant exposure, or bleeding risk
Reperfusion therapy	Thrombolysis or catheter/surgical reperfusion for selected high-risk PE	Evacuation delay increases mortality risk; limited rescue capacity magnifies bleeding consequences	Outcomes of guideline-based reperfusion decisions under conditions of delayed evacuation
Occupational measures and medical thromboprophylaxis	General occupational measures may support mobility and hydration, whereas medical prophylaxis follows established individual VTE and bleeding-risk assessment	Restricted posture, limited mobility, and heat-related dehydration may promote venous stasis, but no direct evidence supports routine medical prophylaxis based solely on underground exposure	Evaluate general occupational measures prospectively; assess IPC, venous-pump devices, and lower-limb ultrasound only in appropriately selected research populations
Dynamic risk assessment	Clinical assessment is generally performed at presentation or when PE is suspected	The temporal relationship between underground exposure and thrombotic outcomes has not been established	First establish exposure–outcome associations, then validate candidate markers, and only thereafter develop and externally validate dynamic warning models
Evacuation planning	Escalation and transfer usually available through hospital systems	Underground access, distance, rescue logistics, and imaging delay change clinical consequences	Develop and prospectively evaluate evacuation thresholds, onsite triage criteria, oxygen-support protocols, transfer monitoring, and destination imaging plans

CTPA, computed tomographic pulmonary angiography; DOACs, direct oral anticoagulants; EIT, electrical impedance tomography; HRV, heart rate variability; IPC, intermittent pneumatic compression; LMWH, low-molecular-weight heparin; PE, pulmonary embolism; SpO₂, peripheral oxygen saturation. The proposed monitoring variables and candidate strategies, including SpO₂, HRV, D-dimer trends, and other exposure-related indicators, remain research hypotheses and have not been validated for PE prediction or management in deep-underground workers. These considerations are not underground-specific clinical recommendations and should not supersede established PE diagnostic or treatment guidelines.

#### 2.4.1 Potential diagnostic ambiguity in deep-underground settings: symptom overlap and occult physiological deterioration

There are currently no comparative studies demonstrating a distinct PE symptom phenotype in deep-underground workers. Nevertheless, diagnostic interpretation may be complicated because several common manifestations of PE overlap with physiological responses to underground exposure. Even in ground-based populations, PE is highly heterogeneous, ranging from asymptomatic or mildly symptomatic disease to exertional dyspnea, syncope, hypoxemia, shock, and sudden death. Chest pain and dyspnea are common clinical clues, whereas hemoptysis is neither necessary nor sufficiently sensitive for diagnosis (3, 4). This heterogeneity may become more pronounced in deep underground environments because symptoms related to PE can overlap with the physiological and subjective effects of underground exposure.

In deep underground work, hypoxia or oxygen fluctuation, high humidity and heat, fatigue, chest discomfort, poor ventilation, and psychological stress may create a background “symptom noise” that obscures early thromboembolic manifestations. Studies in deep underground medicine and confined-space medicine suggest that reduced oxygen availability may not always be readily perceived subjectively, while hypoxia, carbon dioxide accumulation, toxic gases, and airborne particulates may all constitute important atmospheric hazards in enclosed environments (8, 22). In this context, unexplained resting or exertional desaturation, disproportionate tachycardia, abrupt decline in exercise tolerance, increased respiratory rate, syncope or near-syncope, or new perfusion abnormalities may warrant further PE evaluation when they exceed the physiological response expected from environmental exposure alone (9).

In practice, unexplained physiological deterioration should prompt consideration of PE rather than reliance on the classical symptom triad alone, while established diagnostic pathways remain the standard of care. Future studies should evaluate whether portable oxygen saturation and heart rate monitoring, lower-limb venous ultrasound, bedside echocardiography, or EIT-based perfusion monitoring can support interim assessment before definitive imaging becomes available. Established PE diagnostic pathways, including CTPA when indicated, should remain the standard of care (3, 4, 9). The key point is that in deep underground settings, early warning should be based on mismatch between physiological signals and expected environmental response, rather than on classical symptoms alone.

#### 2.4.2 Anticoagulation under deep-underground exposure: pharmacokinetic uncertainty and research priorities

The central issue regarding anticoagulation in deep-underground environments is not whether low-molecular-weight heparin or direct oral anticoagulants become ineffective, but whether compound exposure may alter both drug exposure and the baseline coagulation state. Direct evidence on anticoagulant pharmacokinetics in underground workers is currently lacking. Limited evidence indicates that high-altitude hypoxia can alter the activity of selected drug-metabolizing enzymes and the pharmacokinetics of some cardiovascular drugs (47). Whether these observations are relevant to LMWH or DOAC exposure in underground workers remains unknown.

For PE prevention and treatment, deep underground exposure may shift both sides of the thrombosis–bleeding balance. Prolonged fixed posture, restricted mobility, inadequate hydration, heat-related dehydration, oxygen fluctuation, and psychosocial stress may increase thrombotic risk through venous stasis, hemoconcentration, sympathetic activation, endothelial activation, and procoagulant signaling. At the same time, hypoxia- or inflammation-mediated changes in metabolic enzymes, altered hepatic or renal perfusion, and prerenal dehydration may increase anticoagulant exposure and bleeding risk. These considerations raise the possibility of altered anticoagulant exposure in physiologically nonsteady states, but do not demonstrate that standard LMWH or DOAC dosing becomes unreliable in underground workers (47).

These considerations do not currently justify exposure-based dose adjustment in clinical practice. Rather, they identify pharmacokinetic and pharmacodynamic questions that should be examined in controlled or prospective studies. Anti-Xa activity and direct oral anticoagulant concentration measurements are not routinely required in standard practice and cannot currently be recommended solely on the basis of underground exposure. Their role, if any, should first be examined within prospective pharmacokinetic or pharmacodynamic studies.

#### 2.4.3 Reperfusion decisions under delayed evacuation and research priorities for preventive interventions

In deep underground PE care, the key question is not whether thrombolysis should be used more aggressively, but how established reperfusion decisions should be operationalized when evacuation, imaging confirmation, and advanced rescue resources are delayed. No clinical trial has directly evaluated thrombolysis for PE in deep underground environments. Therefore, current considerations should remain anchored in established PE severity assessment while accounting for evacuation delay and onsite monitoring capacity (3, 4).

When timely evacuation is impossible, reperfusion decisions may become operationally more complex; however, treatment eligibility should remain based on established hemodynamic and bleeding-risk criteria rather than on underground exposure alone. In contrast, for hemodynamically stable intermediate- or low-risk PE, deep underground exposure alone should not lower the threshold for systemic thrombolysis. Anticoagulation, oxygen therapy, circulatory support, continuous monitoring, and urgent transfer should remain the priorities whenever feasible. In patients with trauma, recent surgery, intracranial disease, active bleeding, thrombocytopenia, or coagulation abnormalities, delayed evacuation may increase PE-related mortality risk but also magnify the consequences of thrombolysis-related bleeding.

Preventive approaches in deep-underground settings should distinguish general occupational measures from medical thromboprophylaxis. General measures such as adequate hydration, opportunities for regular movement, periodic interruption of prolonged fixed posture, and ergonomic modification of constrained work positions may help reduce dehydration and venous stasis. However, their effectiveness in preventing VTE among deep-underground workers has not been directly established and should be evaluated in prospective occupational studies.

In contrast, routine medical mechanical prophylaxis, including intermittent pneumatic compression or lower-limb venous pump devices, cannot currently be recommended for underground workers solely on the basis of occupational exposure. Their use should remain governed by established clinical indications, contraindications, and individual VTE and bleeding-risk assessment (48, 49). Future studies may evaluate their feasibility and potential benefit in selected high-risk or temporarily immobilized workers, but underground exposure alone should not constitute an indication for their use. Similarly, routine lower-limb venous ultrasound screening is not supported by direct evidence in underground workers and should currently be regarded as a candidate strategy for prospective research rather than as a standard occupational recommendation.

#### 2.4.4 A staged research pathway toward dynamic risk prediction

Dynamic risk prediction represents a potential long-term research objective rather than an immediately applicable strategy. Its development should be conditional on prior evidence that deep-underground exposure is associated with measurable thrombotic outcomes and that exposure-related variables provide reproducible information beyond established clinical predictors. Research should therefore proceed through three sequential stages.

The first stage should establish the exposure–outcome relationship in prospective, exposure-characterized cohorts of underground workers. Environmental parameters such as oxygen availability, temperature, humidity, atmospheric pressure, particulate or gas exposure, ventilation status, and cumulative underground work duration should be recorded alongside clinically meaningful outcomes, including incident DVT, confirmed PE, hospitalization, bleeding events, and mortality. This stage should determine whether specific underground exposure patterns are associated with thrombotic outcomes after accounting for conventional VTE risk factors. Without such evidence, development of an underground-specific prediction model would be premature.

The second stage should identify reproducible candidate markers and evaluate their incremental value beyond established clinical predictors. Candidate variables may be organized into four domains: environmental exposure; work behavior, including fixed-posture duration and activity level; physiological response, including heart rate, HRV, blood pressure, SpO₂, respiratory rate, temperature, fatigue, and hydration-related indicators; and thrombotic susceptibility, including hemoconcentration, D-dimer, coagulation–fibrinolysis markers, inflammatory or endothelial injury markers, and lower-limb venous ultrasound findings. Environmental sensors may enable time-stamped collection of underground exposure variables (50), whereas wearable or computer vision-based systems may support the assessment of activity, posture, and ergonomic exposure (51). However, these variables should advance to model development only if they are feasible to collect, reproducible across occupational settings, and demonstrate incremental predictive value beyond a clinical-only reference model. Continuous biometric monitoring in underground workplaces also poses substantial implementation challenges. Deep tunnels and complex rock structures may limit wireless communication and real-time data transmission, requiring local data storage, intermittent synchronization, or dedicated underground communication infrastructure. Battery life and sensor durability may be compromised by long shifts, heat, humidity, dust, vibration, and repeated equipment use. Wearable devices may also interfere with protective clothing or work tasks, reducing worker adherence and increasing missing or low-quality data. Importantly, physiological signals such as heart rate, SpO₂, and HRV are nonspecific and may fluctuate with physical exertion, heat stress, fatigue, anxiety, or transient oxygen variation. Poorly calibrated alert thresholds could therefore generate frequent false-positive warnings, producing alarm fatigue, unnecessary work interruption, or evacuation and ultimately reducing acceptance by both workers and employers. Future implementation studies should consequently evaluate data completeness, battery endurance, signal reliability, worker acceptability, false-alarm rates, and operational consequences alongside predictive performance.

Only in the third stage should dynamic warning models be developed and externally validated. Conventional statistical, time-series, or machine-learning approaches could then be compared, with priority given to calibration, interpretability, clinical utility, false-alarm burden, and operational feasibility rather than discrimination alone. Any eventual model should be evaluated through internal and external validation and decision-curve analysis and should be compared directly with established clinical predictors. Its intended output would be an actionable occupational risk state rather than a definitive diagnosis of PE. The thresholds and corresponding responses cannot be specified from the current evidence and would require prospective implementation studies (52–55). Dynamic warning should therefore be viewed as a future surveillance strategy whose development depends on successful completion of the preceding epidemiological and marker-validation stages, rather than as a substitute for established PE diagnostic pathways.

## 3 Conclusion and future perspectives

This review examines deep-underground environments not merely as occupational backgrounds, but as potential modifiers of thrombotic susceptibility that warrant direct investigation. The potential contribution of deep underground exposure is unlikely to be explained by a single factor such as hypoxia, mild hyperbaric pressure, or restricted mobility alone. Instead, it may arise from the convergence of hypoxic sensing, pulmonary vasoconstriction, increased right ventricular load, autonomic readaptation disorder, venous stasis, endothelial activation, and coagulation–inflammation feedback. Within this framework, we therefore propose failed physiological adaptation as a testable mechanistic hypothesis linking compound underground exposure to thrombotic susceptibility, rather than as an established disease mechanism or a distinct form of PE.

The concept of a deep-underground-acquired prothrombotic tendency remains hypothetical and requires empirical validation. At present, direct epidemiological and mechanistic evidence linking deep underground exposure to PE is limited. Most available evidence is indirect and derived from studies of hypoxia physiology, high-altitude medicine, confined environments, occupational health, spaceflight, hemostasis, and conventional VTE pathobiology. This limitation should be explicitly acknowledged. Accordingly, the current evidence is insufficient to conclude that deep-underground exposure independently increases PE risk or that existing ground-based PE tools perform inadequately in underground workers. What the available evidence does support is a set of testable questions: whether underground exposure is associated with measurable prothrombotic changes, whether established PE predictors behave differently in this setting, and whether exposure-related variables provide incremental predictive or clinical value beyond conventional risk factors.

Future work should therefore proceed sequentially: first, prospective exposure-characterized cohorts should establish whether underground exposure is associated with measurable prothrombotic or VTE outcomes; second, candidate exposure and physiological markers should be evaluated for reproducibility and incremental value beyond established predictors; and only thereafter should dynamic warning models be developed and externally validated.
